# Zero birefringence films of pullulan ester derivatives

**DOI:** 10.1038/srep46342

**Published:** 2017-04-18

**Authors:** Takahiro Danjo, Yukiko Enomoto, Hikaru Shimada, Shogo Nobukawa, Masayuki Yamaguchi, Tadahisa Iwata

**Affiliations:** 1 Graduate School of Agricultural and Life Sciences, The University of Tokyo, 1-1-1 Yayoi, Bunkyo-ku, Tokyo 113-8657, Japan; 2 School of Materials Science, Japan Advanced Institute of Science and Technology, 1-1 Asahidai, Nomi, Ishikawa 923-1292, Japan

## Abstract

High-performance films with almost zero-birefringence and zero-wavelength dispersion were succeeded to prepare from pullulan esters derivatives (PLEs) without any additives. Optical transmittance analysis, birefringence measurement of PLE cast film and hot stretched films, and infrared dichroism analysis were conducted to characterize optical properties of PLE films comparing with cellulose triacetate which is commercially used as low-birefringence in optical devices. The aims of this study, characterization of optical properties of pullulan esters, can develop a deep understanding of the fundamental knowing and applicability of polysaccharides. Accordingly, authors believe this paper will open the gate for researches in the application of polysaccharides.

Bio-based plastics from renewable resources are considered eco-friendly. It is hoped they will contribute to a more sustainable society and help prevent global warming. Besides cellulose, which is the most abundant biomass material on earth, there are many kinds of natural polysaccharides with various characteristic structures. Such polysaccharides include xylan and glucomannan, which are extracted from hard and soft woods; chitin and chitosan, which are obtained from crabs and prawns; and curdlan and pullulan, which are synthesized by microorganisms. Natural polysaccharides should be used for the development of new highly functional bio-based polymers, and their characteristic structures should be retained^1^. However, natural polysaccharides are not thermoplastics. Therefore, polysaccharide derivatives such as cellulose ester and ether derivatives are widely used in filter, fiber, and film materials in many areas.

Cellulose triacetate (CTA) is the most widely used material for polarizer protective films in liquid-crystal display devices because it has several attractive properties: high optical transparency, heat resistance, and, in particular, low birefringence. Several methods have been developed to achieve zero birefringence, such as blending with a miscible polymer with intrinsic birefringence of a different sign^2^,^3^,^4^, or blending with low-molecular weight additives, such as anisotropic molecules or birefringence crystals^5^,^6^. For example, whereas the orientation birefringence of pure cellulose acetate propionate (CAP) at a draw ratio of 1.5 was approximately 5 × 10^−4^, this value decreased by half when blended with appropriate additives^7^. On the other hand, when focusing on wavelength dispersion of birefringence in the visible light region, it is prefer that absolute value of birefringence increase with increasing wavelength of light, which is so-called extraordinary dispersion, in order to eliminate the dependence of the phase retardation on the wavelength of light. Although orientation birefringence of stretched CTA film shows ordinary wavelength dispersion, i.e. absolute value of birefringence decrease with increasing wavelength of light, extraordinary wavelength dispersion could be attained with cellulose mixed ester derivatives such as cellulose acetate propionate or acetate butyrate (CAB)^8^,^9^. A film with zero birefringence and quite small and extraordinary wavelength dispersion is desirable for a protective film that does not change the polarization state and wavelength of transmitted light. Herein, we report films with almost zero birefringence and zero wavelength dispersion prepared from pullulan esters (PLEs).

Pullulan (PL) is a linear polysaccharide that is extracellularly produced by strains of the fungus *Aureobasidium pullulans*. Whereas cellulose consists of β-(1 → 4)-linked glucose units, pullulan comprises periodically linked α-(1 → 4) and α-(1 → 6) glucose units that form a step-like chemical structure (Fig. 1)^10^,^11^. PL has valuable properties such as water-soluble, non-toxic, edible, and film-forming abilities, and is widely used in food and biomedical fields^11^,^12^,^13^. For further functionalization of PL, several studies on chemical modifications of PL have been reported, such as succinylation^14^, sulfation^15^, or modification with isocyanates^16^ or cholesteryl group^17^. In particular, acylation of hydroxyl groups of PL with carboxylic acids is one of popular and simple method to obtain hydrophobized derivatives with thermo-plasticity or organic solvent solubility like PL acetate^18^,^19^,^20^. Recently, our research group reported thermal and mechanical properties of pullulan acyl derivatives with acyl carbon number of 2–12, which have non-crystalline properties unlike CTA^21^. However, there is no report about optical properties for pullulan derivatives. In the present study, we investigated PLE films with small acyl carbon number (*N*) values such as pullulan acetate (PLAc: *N* = 2), pullulan propionate (PLPr: *N* = 3) and pullulan acetate propionate (PLAcPr: *N* = 2,3) with high transparency. Furthermore, we determined the optical anisotropy of each PLE and compared it with that of CTA.

## Optical transmittance of PLE and CTA cast films

**Figure 2:**
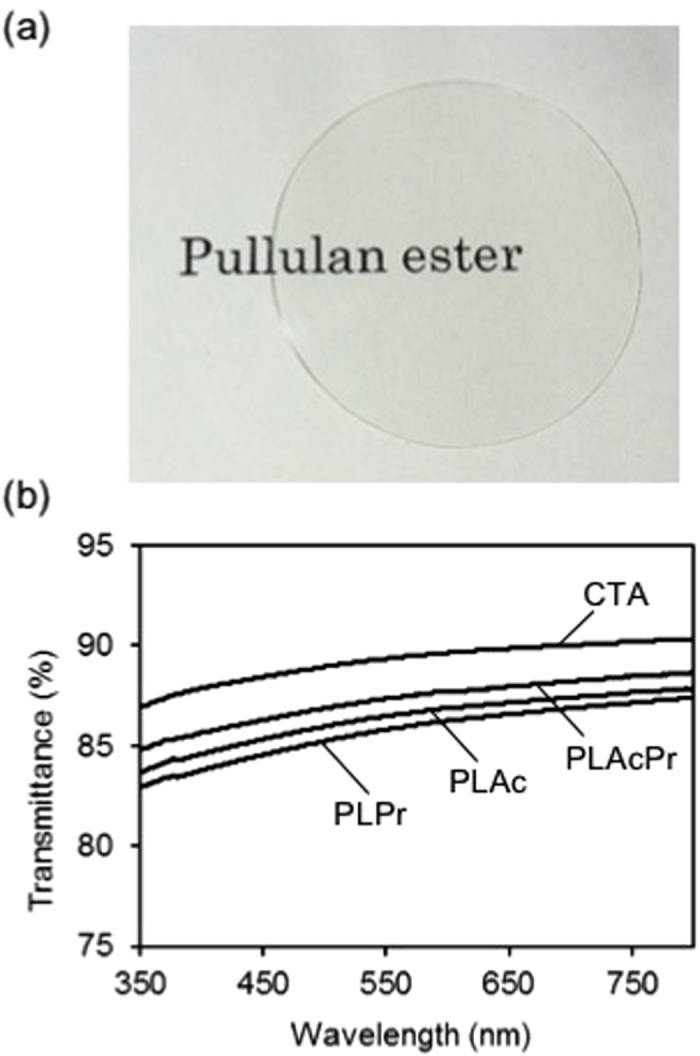
(**a**) Representative image of solvent-cast film of pullulan propionate (PLPr); (**b**) Optical transmittance spectra of pullulan ester (PLE) and cellulose triacetate (CTA) cast films.

Figure 2(a) shows a representative image of a PLPr cast film. We used solvent casting to form highly transparent films from all the PLEs. Figure 2(b) shows the optical transmittance spectra of the cast films. Each PLE film had a transmittance percentage within the range 83–88% in the visible light wavelength range (380–750 nm). Although the transmittance percentage values of the PLE films were slightly lower than that of CTA (87–90%), the values were still high considering interface reflection (i.e., Fresnel reflection), indicating that PLEs absorb little visible light and are suitable for transparent film materials. Each PLE had an average refractive index (*n*_ave_) within the range 1.46–1.48 (Table 1), which was almost the same as that for CTA (1.47–1.48)^22^.

## Out-of-plane birefringence of PLE and CTA cast films

Three refractive indices, *n*_x_, *n*_y_, and *n*_z_, along three principal axes must be taken into consideration when assessing the optical anisotropy of films. The *x*-axis is the direction with the maximum refractive index within the film plane, the *y*-axis is the direction perpendicular to the *x*-axis within the film plane, and the *z*-axis is normal to the *x–y* plane. In polymer films, refractive index anisotropy mainly results from the orientation of the polymer chains, and is called orientation birefringence (Δ*n*(λ)). Using the Herman’s orientation function, orientation birefringence is expressed by the following equation (1):


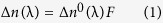


where λ, 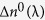, and *F* are the wavelength of light, the intrinsic birefringence of the polymer, and the orientation function, respectively. The intrinsic birefringence is the asymmetry in the refractive indices that originates from the chemical structure of the repeating units of the polymers^2^,^9^.

As is well known, the solvent-casting method, which is used to produce commercial CTA optical films, provides films without molecular orientation in the film plane, i.e., *n*_x_ = *n*_y_. Three-dimensional assessment of refractive indices is then required to characterize birefringence properties.

**Figure 3:**
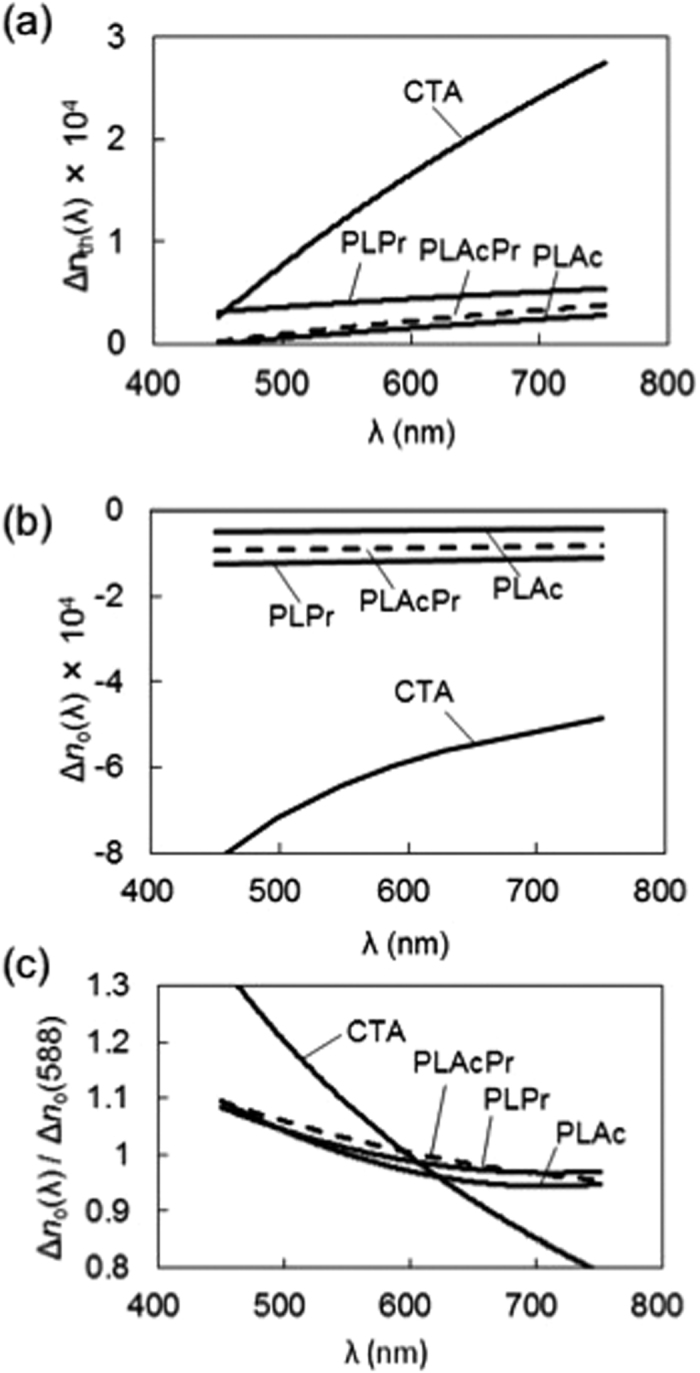
(**a**) Out-of-plane birefringence of pullulan ester (PLE) and cellulose triacetate (CTA) cast films; (**b**) Orientation birefringence of PLE and CTA hot-stretched films; and (**c**) Normalized orientation birefringence for PLE and CTA hot-stretched films.

Figure 3(a) shows the change in the out-of-plane birefringence (Δ*n*_th_) with the wavelength of light of the PLE cast films compared with that of the CTA cast film. As reported previously^8^, the CTA cast film had a positive birefringence (*n*_x_, *n*_y_ > *n*_z_), which increased with increasing wavelength, i.e. extraordinary wavelength dispersion. In general, molecular orientation in solvent-cast films is caused by the compressional stress resulting from solvent evaporation^23^. Because CTA molecules have intrinsic birefringence that is associated with the anisotropic polarizability of the acetyl group, CTA molecular orientation in cast film results in birefringence.

PLE cast films also show positive birefringence and extraordinary dispersion, although their absolute birefringence values are quite small compared with that of CTA. This result is probably caused by the difference in molecular structure between pullulan and cellulose, and by the associated difference in molecular mobility during film formation. The Δ*n*_th_ value of PLAcPr is in between those of PLAc and PLPr, indicating that mixed esterification of pullulan could result in a birefringence that is intermediate between that of the homo-esters. We carried out orientation birefringence measurements to investigate further the relationship between polymer orientation and birefringence.

## Orientation birefringence of oriented PLE films

Table 2 shows the drawing conditions for each PLE film. In this study, the drawing temperature (*T*_draw_) was set within a specific range where the tensile storage modulus (*E*′) was in the range 100–10 MPa, with consideration for the *T*_g_ of each PLE. The *T*_draw_ values were higher than the *T*_g_ values of the PLEs, which were defined as the peak temperatures associated with the tensile loss modulus *E*″ from dynamic mechanical analysis (DMA). The *T*_draw_ values for the films were ultimately decided by taking temperatures that provided almost the same tensile stress values (approximately 0.25–0.28 MPa) at a draw ratio of 1.5, based on the stress-optical law; the orientation birefringence Δ*n*_o_ of a polymer is proportional to the stress *σ*, as following equation (2):


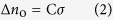


where C is the stress-optical coefficient.

Figure 3(b) shows the change in orientation birefringence (Δ*n*_o_) with wavelength of the PLE oriented films and CTA oriented film (*T*_draw_ = 215 °C, tensile stress at 1.5 strain = 14 MPa). As reported previously^9^,^24^, the CTA oriented film had a negative birefringence with ordinary wavelength dispersion; the absolute value of birefringence decreased with increasing wavelength. The PLE oriented films also had negative orientation birefringence, but the absolute values of birefringence were noticeably lower than the birefringence of the CTA film, and little wavelength dispersion was observed. PLAcPr had a Δ*n*_o_ value that was intermediate between those of the homo-esters. These results are similar to those of the out-of-plane birefringence of the cast films. Low wavelength dispersion of birefringence of PLEs is demonstrated by taking normalized orientation birefringence (Fig. 3(c)). This figure demonstrates that the PLE oriented films had essentially smaller wavelength dispersion of orientation birefringence values than the CTA film, which can be explained by the difference in chemical structure. However, the low Δ*n*_th_ and Δ*n*_o_ of the PLE films were probably caused not only by the differences in chemical structure, but also by the low degree of orientation of the pullulan backbone in the in-plane direction of the cast film or in the stretched direction of the oriented film. The low orientation of the PLE molecules in the film is suggested by the smaller tensile stress values of the PLEs (1.9–2.2 MPa) compared with CTA (14 MPa), according to the stress-optical law.

## Infrared dichroism of PLE and CTA stretched films

**Figure 4:**
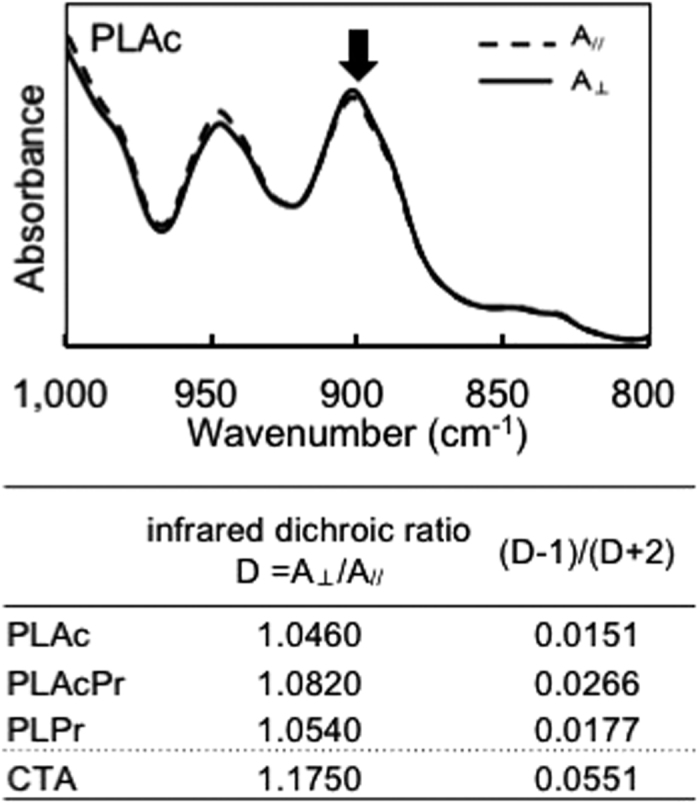
Absorbance of polarized infrared by oriented pullulan acetate (PLAc) film, and dichroic ratios of pullulan esters (PLEs) and cellulose triacetate (CTA).

To clarify the stretching state of the PLEs in the oriented film, the infrared dichroic ratio was measured by polarized Fourier transform infrared spectroscopy (FT-IR). The infrared dichroism of polysaccharide derivatives can provide information about the orientation state of the polysaccharide backbone^7^. In this study, we used the band located at approximately 885–905 cm^−1^, which is attributed to skeletal mode vibrations of the glucopyranose ring^25^,^26^. Figure 4 shows a representative FT-IR spectrum of a stretched PLAc film. We found that the absorbance of the polarized IR beam in the direction perpendicular to the stretching direction (A_⊥_) was slightly stronger than that parallel to the stretching direction (A_//_), indicating that the electric vector of the focused band was perpendicular to the stretching direction.

The relationship between the dichroic ratio *D* (=A_⊥_/A_//_) and the orientation function (*F*) is expressed as following equation (3):


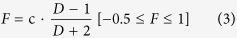


where *c* is a correction for the inclination of the transition moment direction of the infrared absorption band from the polymer chain axis. In this study, the orientation state of each polysaccharide ester was simply discussed by comparing *D* and (*D* − 1)/(*D* + 2) values under the assumption that the *c* value remained constant.

The *D* and (*D* − 1)/(*D* + 2) values for each PLE and for the CTA are shown in Fig. 4. The absolute values of (*D* − 1)/(*D* + 2) for the PLEs (0.015–0.026) were smaller than for the CTA (0.055). This result suggests that the degree of orientation of the PLE chain in the oriented films was smaller than in the CTA film, which is supported by low tensile strength of PLE films under hot stretching. Thus, it is supposed that the stress relaxation of the PLE molecules during film formation was rapid, resulting in PLE films with quite low birefringence. The rapid orientation relaxation could be caused by the unique step-like structure of pullulan molecules having two kinds of α-glycosylic linkage while cellulose has liner β-glycosylic linkage.

## Conclusion

High-performance films with high optical transparency, almost zero birefringence and zero wavelength dispersion were prepared from natural polysaccharide derivatives. Solvent-cast films of PLE had a high optical transmittance percentage within the range 83–88% in visible light region, and had quite low out-of-plane birefringence compared with CTA. Orientation birefringence measurement and infrared dichroism of PLE thermally stretched films demonstrated that low birefringence value of thermally stretched PLE films originated from small degree of orientation of PLE molecules, indicating fast orientation relaxation of PLE during film formation. On the other hand, it was also suggested PLE films had essentially small wavelength dispersion of birefringence. Based on this result, bio-based polymers with outstanding properties and functions could be produced from natural polysaccharides maintaining characteristic chemical structures without saccharification, and it is found the possibility that a new material development without depending on fossil resources.

## Methods

### Materials

Powdered pullulan (PL) was purchased from Tokyo Chemical Industry Co., Ltd, Japan. Cotton lint cellulose was purchased from Walmart Stores, Inc. Trifluoroacetic anhydride (TFAA), acetic acid, propionic acid, and all other reagents were purchased from Wako Pure Chemical Industries, Ltd, Japan, and were used without further purification.

### Preparation of pullulan esters and cellulose acetate

PLEs and CTA were prepared according to the procedure described in our previous study^21^. A premixed solution of TFAA (100 mL) and acetic or propionic acid (100 mL), which had been stirred at 50 °C for 20 min, was immediately added to the freeze-dried PL (2.0 g) in a flask, and stirred at 50 °C for 2 h. The obtained homogeneous solution was cooled to room temperature, poured into a mixed solution comprising water (500 mL) and methanol (1000 mL), and stirred for 10–20 min to complete precipitation. The precipitate was filtered, washed with methanol, dissolved in trichloromethane (150 mL), and reprecipitated in methanol (1000 mL), before final filtering, washing with methanol, and drying *in vacuo* to produce the solid PL ester. The degree of substitution (DS) of the acetyl group (DS_Ac_) and propionyl group (DS_Pr_) were calculated from the ratio of the integrated area of the methyl protons of the acyl groups to the ring protons of glucose, as follows: DS = ([CH_3_]/3)/([ring-H]/7). Ac and Pr represent acetyl and propionyl groups, respectively. CTA was synthesized in the same way using cotton lint cellulose (Table 1).

### Nuclear magnetic resonance (NMR) measurements

^1^H-NMR spectra of the PLEs were obtained by using JEOL JNM-A500 FT-NMR (500 MHz) equipment at 25 °C. Chemical shifts (δ in ppm) were expressed relative to the resonance of tetramethylsilane (TMS) (δ = 0). Deuterated trichloromethane (CDCl_3_) was used as the solvent.

### Gel permeation chromatography (GPC) measurements

The number- and weight-average molecular weights (*M*_n_ and *M*_w_) and the polydispersity index (*M*_w_/*M*_n_) values of the PLEs were estimated by GPC (CBM-20A, DGU-20A3, LC-6AD, SIL-20ACHT, CTO-20A, RID-10A, Shimadzu) in trichloromethane at 40 °C. Shodex columns (K-806M, K-802) were used, and the flow rate was 0.8 mL/min. A calibration curve was obtained using polystyrene standards (Shodex).

### Preparation of solvent-cast PLE films

Films of PLEs were prepared using the solvent-casting method. PLE was dissolved in trichloromethane at a concentration of 4 wt%. The solution (6.4 g) was poured into a polytetrafluoroethylene (PTFE) petri dish (50-mm diameter) with a flat bottom, and evaporated at room temperature for 3 h. The obtained film was dried *in vacuo* (<0.01 MPa) for 24 h. The thickness of the obtained films was approximately 90 μm. The average refractive index (*n*_ave_) of each PLE cast film was measured using an Abbe refractometer ATAGO 1 T (ATAGO). The *n*_ave_ value was used to determine the birefringence.

### Optical transmittance of PLE cast films

The optical transmittance of the PLE cast films was determined using a U-2910 spectrophotometer (Hitachi). The scan range was 250–800 nm, and the total transmittance was detected at room temperature.

### Out-of-plane birefringence of PLE cast films

The optical anisotropy of the PLE cast films was determined by out-of-plane birefringence measurements using an optical birefringence analyzer KOBRA-WPR (Oji Scientific Instruments). Prior to examination, the PLE films were placed overnight in a temperature- and humidity-controlled chamber (IG420, Yamato) at 25 °C and 50% relative humidity (RH) to avoid the effects of moisture on the optical properties. The retardation in the vertical direction of the film (*R*_th_) was determined by retardation measurements at an oblique incidence angle of 40° as a function of wavelength by changing the color filters. The corresponding birefringence value (out-of-plane birefringence) Δ*n*_th_ was calculated from the film thickness (*d*) measured using a micrometer.

*R*_th_ was defined by the following equation (4):


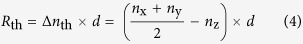


where *n*_x_, *n*_y_, and *n*_z_ are the refractive indices in the three principal axes^8^.

The dependence of the phase retardation of transmitted light on incidence angle, and the optimum condition to eliminate the viewing angle dependence are described in some literatures^27^,^28^.

### Preparation of oriented PLE films

Optical anisotropy induced by polymer chain orientation was determined by measuring the birefringence of the oriented films. The uniaxial oriented films were prepared by hot drawing the rectangular cast films (10 × 20 mm) at a constant stretching rate of 0.5 mm/s in a dynamic mechanical analyzer S100-DVE3 (UBM). The initial distance between the clamps was 10 mm and the draw ratio was 1.5. The drawing temperature (*T*_draw_) was set above the glass transition temperature (*T*_g_), and the tensile storage modulus *E*′ was in the range 100–10 MPa observed using a dynamic mechanical analyzer Rheogel-E4000 (UBM) at 10 Hz (Table 2). After stretching, the film was immediately quenched in a stream of cold air to avoid relaxation of the molecular orientation. The oriented films were stored in a chamber at 25 °C and 50% RH before further analysis.

### Orientation birefringence of oriented PLE films

The influence of polymer chain orientation on the optical anisotropy of the films was determined by orientation birefringence measurement of the stretched PLE films using KOBRA-WPR equipment (Oji Scientific Instruments). The in-plane retardation (*R*_in_) and the orientation birefringence (Δ*n*_o_) were defined by the following equation (5):


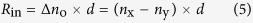


where *n*_x_ is the refractive index in the stretching direction and *n*_y_ is the refractive index in the vertical direction from the *x* plane.

### Infrared dichroism of oriented PLE films

The infrared dichroism of the oriented PLE films was determined using an FT-IR spectrometer NICOLET 6700 (Thermo Fisher Scientific Inc.) equipped with a KRS-5 polarizer. The absorbance of the pyranose ring (885–905 cm^−1^) 7 was measured using a linearly polarized IR beam with an electric vector parallel or perpendicular to the stretching direction. Absorbance in the parallel (A_//_) and perpendicular (A_⊥_) directions were measured, and the dichroic ratio *D* (=A_⊥_/A_//_) was calculated.

## Additional Information

**How to cite this article**: Danjo, T. *et al*. Zero birefringence films of pullulan ester derivatives. *Sci. Rep.* **7**, 46342; doi: 10.1038/srep46342 (2017).

**Publisher's note:** Springer Nature remains neutral with regard to jurisdictional claims in published maps and institutional affiliations.

## Figures and Tables

**Figure 1:**
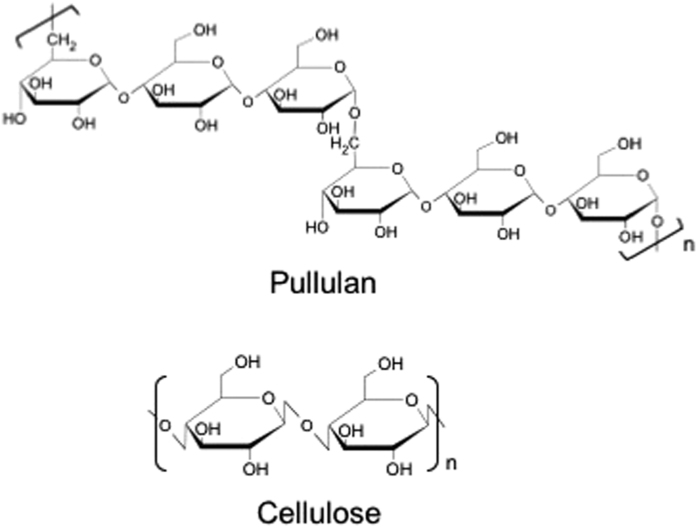
Chemical structures of pullulan and cellulose.

**Table 1:** Characteristics of pullulan esters (PLEs).

Sample	Abbreviation	DS^a^		*M*_w_ (×10^5^)^b^	*M*_n_ (×10^5^)^b^	*M*_w_/*M*_n_	*n*_ave_^c^	*T*_g_ (°C)^d^
		DS_Ac_	DS_Pr_					
Pullulan acetate	PLAc	3.0	—	3.16	1.29	2.45	1.48	164
Pullulan acetate propionate	PLAcPr	1.6	1.4	3.17	1.44	2.21	1.47	139
Pullulan propionate	PLPr	—	3.0	3.03	1.54	1.97	1.46	118

^a^Calculated by ^^1^^H-NMR spectra.

^b^Estimated by GPC using polystyrene standards.

^c^Measured by Abbe’s refractometer.

^d^Observed by DSC.

**Table 2:** Preparation conditions for pullulan ester (PLE) oriented films.

	Temperature (°C)*		*T*_draw_ (°C)	Stress at 1.5 strain (MPa)
	*E*′ = 100 MPa	*E*′ = 10 MPa		
PLAc	159	169	168	0.25
PLAcPr	149	156	149	0.27
PLPr	127	133	127	0.28

*Observed by DMA.

## References

[b1] Iwata T. Biodegradable and bio-based polymers: future prospects of eco-friendly plastics. Angewandte Chemie 54, 3210–3215, doi: 10.1002/anie.201410770 (2015). 25583677

[b2] Saito H. & Inoue T. Chain Orientation and Intrinsic Anisotropy in Birefringence-Free Polymer Blends. J Polym Sci Pol Phys 25, 1629–1636, doi: 10.1002/polb.1987.090250806 (1987).

[b3] Hahn B. R. & Wendorff J. H. Compensation Method for Zero Birefringence in Oriented Polymers. Polymer 26, 1619–1622, doi: 10.1016/0032-3861(85)90273-3 (1985).

[b4] Yamaguchi M. & Masuzawa K. Birefringence control for binary blends of cellulose acetate propionate and poly(vinyl acetate). European Polymer Journal 43, 3277–3282, doi: 10.1016/j.eurpolymj.2007.06.007 (2007).

[b5] Tagaya A., Iwata S., Kawanami E., Tsukahara H. & Koike Y. Zero-birefringence polymer by the anisotropic molecule dope method. Appl Opt 40, 3677–3683, doi: 10.1364/AO.40.003677 (2001). 18360398

[b6] Tagaya A., Ohkita H., Mukoh M., Sakaguchi R. & Koike Y. Compensation of the birefringence of a polymer by a birefringent crystal. Science 301, 812–814, doi: 10.1126/science.1086966 (2003). 12907795

[b7] Yamaguchi M., Iwasaki T., Okada K. & Okamoto K. Control of optical anisotropy of cellulose esters and their blends with plasticizer. Acta Materialia 57, 823–829, doi: 10.1016/j.actamat.2008.10.018 (2009).

[b8] Songsurang K. . Optical anisotropy in solution-cast film of cellulose triacetate. Cellulose 20, 83–96, doi: 10.1007/s10570-012-9807-0 (2013).

[b9] Yamaguchi M. . Extraordinary Wavelength Dispersion of Orientation Birefringence for Cellulose Esters. Macromolecules 42, 9034–9040, doi: 10.1021/ma901676j (2009).

[b10] Bender H., Lehmann J. & Wallenfels K. Pullulan, ein extracelluläres Glucan von Pullularia pullulans. Biochimica et Biophysica Acta 36, 309–316, doi: 10.1016/0006-3002(59)90172-6 (1959). 13798777

[b11] Leathers T. D. Biotechnological production and applications of pullulan. Appl Microbiol Biotechnol 62, 468–473, doi: 10.1007/s00253-003-1386-4 (2003). 12830329

[b12] Singh R. S., Saini G. K. & Kennedy J. F. Pullulan: Microbial sources, production and applications. Carbohydrate polymers 73, 515–531, doi: 10.1016/j.carbpol.2008.01.003 (2008). 26048217

[b13] Farris S., Unalan I. U., Introzzi L., Fuentes-Alventosa J. M. & Cozzolino C. A. Pullulan- Based Films and Coatings for Food Packaging: Present Applications, Emerging Opportunities, and Future Challenges. J Appl Polym Sci 131, doi: 10.1002/app.40539 (2014).

[b14] Bruneel D. & Schacht E. Chemical Modification of Pullulan.3. Succinoylation. Polymer 35, 2656–2658, doi: 10.1016/0032-3861(94)90395-6 (1994).

[b15] Mihai D., Mocanu G. & Carpov A. Chemical reactions on polysaccharides I. Pullulan sulfation. European Polymer Journal 37, 541–546, doi: 10.1016/S0014-3057(00)00142-7 (2001).

[b16] Shibata M., Asahina M., Teramoto N. & Yosomiya R. Chemical modification of pullulan by isocyanate compounds. Polymer 42, 59–64, doi: 10.1016/S0032-3861(00)00321-9 (2001).

[b17] Akiyoshi K., Deguchi S., Moriguchi N., Yamaguchi S. & Sunamoto J. Self-Aggregates of Hydrophobized Polysaccharides in Water-Formation and Characteristics of Nanoparticles. Macromolecules 26, 3062–3068, doi: 10.1021/ma00064a011 (1993).

[b18] Motozato Y., Ihara H., Tomoda T. & Hirayama C. Preparation and Gel-Permeation Chromatographic Properties of Pullulan Spheres. J Chromatogr 355, 434–437, doi: 10.1016/S0021-9673(01)97349-2 (1986).

[b19] Tezuka Y. Pullulan nonaacetate: Assignment of chemical shifts of the acetyl protons and acetyl carbonyl carbons by 2D-NMR spectroscopy. Carbohyd Res 305, 155–161, doi: 10.1016/S0008-6215(97)10036-2 (1997).

[b20] Teramoto N. & Shibata M. Synthesis and properties of pullulan acetate. Thermal properties, biodegradability, and a semi-clear gel formation in organic solvents. Carbohydrate polymers 63, 476–481, doi: 10.1016/j.carbpol.2005.10.008 (2006).

[b21] Enomoto-Rogers Y., Iio N., Takemura A. & Iwata T. Synthesis and characterization of pullulan alkyl esters. European Polymer Journal 66, 470–477, doi: 10.1016/j.eurpolymj.2015.03.007 (2015).

[b22] Mark J. E. Polymer data handbook. 2nd edn, (Oxford University Press, 2009).

[b23] Croll S. G. The origin of residual internal stress in solvent-cast thermoplastic coatings. J Appl Polym Sci 23, 847–858, doi: 10.1002/app.1979.070230319 (1979).

[b24] Yamaguchi M., Abd Manaf M. E., Songsurang K. & Nobukawa S. Material design of retardation films with extraordinary wavelength dispersion of orientation birefringence: a review. Cellulose 19, 601–613, doi: 10.1007/s10570-012-9660-1 (2012).

[b25] Kaputskii V. E., Komar V. P. & Skornyakov I. V. Infrared spectra and structure of cellulose phosphates. J Appl Spectrosc 48, 176–179, doi: 10.1007/bf00661996 (1988).

[b26] Liang C. Y. & Marchessault R. H. Infrared spectra of crystalline polysaccharides. II. Native celluloses in the region from 640 to 1700 cm.1. Journal of Polymer Science 39, 269–278, doi: 10.1002/pol.1959.1203913521 (1959).

[b27] Yeh P. & Gu C. Optics of Liquid Crystal Displays. (Wiley Publishing, 2009).

[b28] Hwang J. . Single layer retarder with negative dispersion of birefringence and wide field-of-view. Opt Express 24, 19934–19939, doi: 10.1364/OE.24.019934 (2016). 27557268

